# Measles immunity and immunosuppression

**DOI:** 10.1016/j.coviro.2020.08.002

**Published:** 2021-02

**Authors:** Diane E Griffin

**Affiliations:** W. Harry Feinstone Department of Molecular Microbiology and Immunology, Johns Hopkins Bloomberg School of Public Health, Baltimore, MD 21205, USA

## Abstract

Effects of measles on the immune system are only partially understood. Lymphoid tissue is a primary site of measles virus (MeV) replication where CD150 is the receptor for infection of both B and T cells. Lymphocyte depletion occurs during the acute phase of infection, but initiation of the adaptive immune response leads to extensive lymphocyte proliferation, production of MeV-specific antibody and T cells, the rash and clearance of infectious virus. Viral RNA persists in lymphoid tissue accompanied by ongoing germinal center proliferation, production of antibody-secreting cells, functionally distinct populations of T cells and antibody avidity maturation to establish life-long immunity. However, at the same time diversity of pre-existing antibodies and numbers of memory and naive B cells are reduced and susceptibility to other infections is increased.

## Highlights

• Measles virus replication in lymphoid tissue uses CD150 as a B and T cell receptor.
• Persistent RNA in lymphoid tissue is associated with generation of durable immunity.
• Germinal centers and numbers of peripheral Tfh cells and antibody-secreting cells increase for months.
• Increased susceptibility to other infections continues for years after apparent recovery.
• Memory and naïve B cells are depleted and diversity of pre-existing antibodies is reduced.

**Current Opinion in Virology** 2021, **46**:9–14

This review comes from a themed issue on **Viral immunology**

Edited by **Allan J Zajac** and **Annette Oxenius**

For complete overview of the section, please refer the article collection – Viral Immunology (2021) and https://www.sciencedirect.com/topics/medicine-and-dentistry/viral-immunology

Available online 4th September 2020

**https://doi.org/10.1016/j.coviro.2020.08.002**

1879-6257/© 2020 The Author(s). Published by Elsevier B.V. This is an open access article under the CC BY license (http://creativecommons.org/licenses/by/4.0/).

## Introduction

Measles is a systemic rash disease that is an increasing cause of morbidity and mortality worldwide despite the availability of a safe and effective live attenuated virus vaccine [1,2]. Infection with measles virus (MeV), the causative agent of measles, is initiated in the respiratory tract, rapidly spreads to lymphoid tissue and has profound short and long-term effects on the immune system. For instance, MeV infection increases susceptibility to other infections that are responsible for most of the measles-associated acute mortality [3,4,5^•^], can trigger autoimmune encephalomyelitis [6] and induces lifelong protective immunity [7,8]. Because macaques develop a disease very similar to human measles, investigations aimed at understanding the immunopathogenisis of measles have focused on experimentally infected macaques as well as naturally infected children [9, 10, 11].

MeV is an enveloped negative sense RNA virus that belongs to the Morbillivirus genus of *Paramyxoviridae*. MeV encodes two surface glycoproteins, hemagglutinin (H) and fusion (F); four internal proteins, matrix (M), nucleocapsid (N), phosphoprotein (P) and large polymerase (L); and two nonstructural regulatory proteins, C and V. H is on the surface of the virion as a dimer of dimers in non-covalent association with F and is the attachment protein for cell entry and target for neutralizing antibody. H can interact with several receptors: CD46 is a complement regulatory protein expressed on all nucleated cells, but used mainly by vaccine strains of MeV. Nectin 4 is an adherens junction protein expressed on the basolateral surface of epithelial cells and used by both vaccine and wild type (WT) strains of MeV. CD150/signaling lymphocyte activation molecule (SLAM) is an immunoregulatory protein expressed on activated lymphocytes, monocytes and dendritic cells used by both vaccine and WT viruses and is the primary receptor for WT MeV infection of lymphoid tissue and systemic virus spread [10,12].

## Measles virus replication in lymphoid tissue

After initiation of infection in the respiratory tract, MeV is transported to the draining lymph node (LN) where it infects CD150/SLAMF1-expressing cells. CD150 is a glycosylated immunoglobulin superfamily protein that was the first member (F1) of the six-member SLAM subfamily of CD2 transmembrane proteins. Most SLAM family proteins have an extracellular segment that consists of two immunoglobuin-like domains (one variable (V)-like and one constant (C)-like), a transmembrane and a cytoplasmic domain. The V-like extracellular domain is a self ligand that binds SLAM proteins on other cells in homotypic or heterotypic cell interactions [13, 14, 15]. The cytoplasmic domain has immunoreceptor tyrosine-based switch motifs (ITSMs) that are docking sites for SH2 domain-containing adaptor, tyrosine kinase and tyrosine and inositol phosphatase proteins that mediate and regulate SLAM signaling [16,17].

The MeV receptor SLAMF1 is constitutively expressed on immature thymocytes, naïve and memory B cells, memory T cells and is induced during activation of naïve T cells [18,19]. In dendritic cells and macrophages SLAMF1 is localized to endocytic recycling vesicles and trafficked to the surface with Raf-1 and ERK-induced activation of acidic sphingomyelinase in the vesicles [15,20]. SLAMF1 is a dual function co-stimulatory molecule and interaction of the ITSM in the cytoplasmic domain with SLAM adaptor protein (SAP) determines whether SLAM transmits a positive or negative signal in T cells [16,21]. SAP, first identified as a T cell protein mutated in X-linked lymphoproliferative disease, binds both phosphorylated and unphosphorylated ITSMs to regulate recruitment of phosphatases SHP-1 and SHP-2 and control signaling [16]. SLAM promotes TCR-induced CD28-independent proliferation of memory T cells, Th1 differentiation and IFN-γ production of naïve T cells, IL-17 production, proliferation and cytotoxicity of CD8^+^ T cells, and proliferation and antibody secretion by B cells [21, 22, 23, 24].

H interaction with SLAMF1 or other cellular receptors induces a conformational change in F that leads to fusion of the viral envelope with the plasma membrane and initiation of infection with delivery of the genome into the cell cytoplasm. H also interacts with toll-like receptor (TLR) 2, a pathogen recognition receptor expressed on most immune cells and epithelial cells that signals through adaptor proteins MyD88 and IRAK4 to activate NF-κB, induce transcription of mRNAs for IL-6 family member proteins, IL-1β and TNFα and increase expression of SLAM [25]. The effect of H-induced SLAMF1 or TLR2 signaling on virus replication in lymphocytes or on immune responses to MeV is not known.

**Figure 1:**
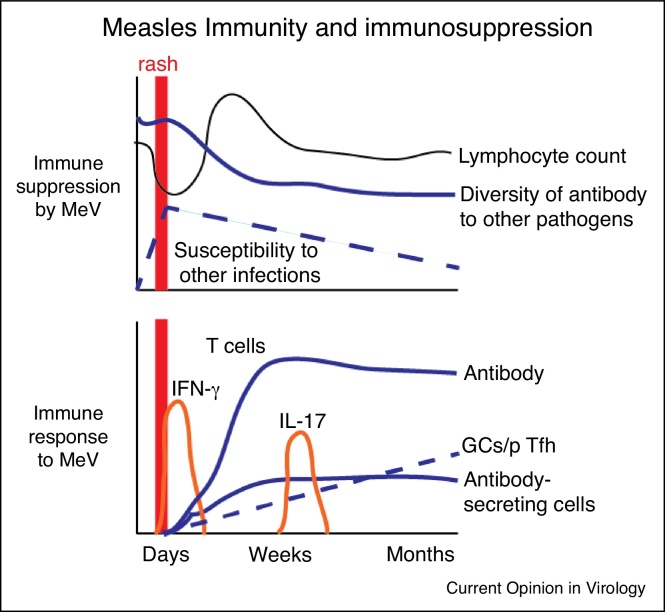
Diagrammatic representation of the dual effects of measles virus (MeV) infection on the immune system: immune suppression with a prolonged increase in susceptibility to other infections (top panel) and induction of a vigorous immune response to MeV that results in life-long immunity to re-infection (lower panel). GC – germinal center; pTfh – peripheral T follicular helper cells.

Immune cells expressing SLAMF1 in lymphoid tissue are the main sites of virus amplification and fuel the viremia and systemic spread of MeV [10]. Virus replication in lymphoid cells has been studied *ex vivo* in thymus organ cultures, tonsil explants and peripheral blood mononuclear cells (PBMCs) and has shown preferential replication in double-positive thymocytes, B cells and memory T cells consistent with SLAMF1 expression [26, 27, 28, 29]. *In vivo,* there is extensive replication of MeV in B and T cells in blood and lymphoid tissues with a higher percentage of MeV-positive B cells than T cells. Replication occurs in both naïve and memory B cells, but primarily in memory T cells [29,30,31^•^,32^••^]. MeV infection can induce lymphocyte cell death and leukopenia commonly accompanies the viremia [29,33,34]. Lymphocyte depletion is followed by a rapid rebound in cell numbers with immune-mediated lymphocyte activation and proliferation as well as an increase in output of cells from the thymus [35,36]. In LNs, this phase of B and T cell depletion is followed at the time of the rash by LN enlargement due to repopulation with proliferating lymphocytes [29]. B cell follicles expand and germinal centers increase in number and continue to produce MeV-specific antibody-secreting cells (ASCs) for several months after recovery [37^••^] (Figure 1).

The effects of cell depletion and proliferation change the relative representation of subtypes of immune cells in circulation over time [31^•^,^38^]. In general, naïve T cells and memory B cells are decreased while activated and memory T cells and regulatory T cells are increased after recovery compared to before infection [31^•^]. Several waves of functionally distinct MeV-specific T cells appear in circulation during recovery and cytokine production shifts from interferon (IFN)-γ to IL-4, IL-10 and IL-17 [39,40]. MeV-specific ASCs are found in blood as the rash fades and then continue to be produced for several months after recovery [37^••^] (Figure 1).

## Immune-mediated clearance

MeV infection is clinically inapparent during the incubation period when virus is actively replicating in lymphoid tissue and spreading systemically. Innate responses are not well defined with evidence primarily of inflammasome (IL-1β, IL-18) and NF-κB (IL-6), rather than type I IFN pathway activation, but these responses do not prevent virus replication and dissemination [11,41]. Clearance is dependent on the adaptive immune response. The maculopapular rash that appears 10–14 days after infection is a manifestation of the cellular immune response to infection with lymphocyte infiltration into sites of virus replication in skin epithelial cells [42]. MeV-specific IFN-γ-producing T cells and IgM antibodies are detectable in blood as the rash is fading and infectious virus is cleared within a week after appearance of the rash. MeV-specific IgM antibodies provide the primary diagnostic test for confirmation of a diagnosis of measles. Although antibody is likely to contribute, MeV-specific T cell responses are required for virus clearance [43,44].

Clinical evidence of the importance of cellular immunity to MeV for virus clearance comes from observations of the outcome of infection in immunocompromised children. Congenital inability to produce antibodies allows recovery from measles, while defects in T-lymphocyte function can lead to fatal progressive pulmonary or neurologic disease [45, 46, 47, 48]. The cellular immune response to MeV infection includes activation of CD4^+^ and CD8^+^ T lymphocytes that evolve functionally during and after recovery [38]. The predominant initial cellular response important for control and clearance of infectious virus is characterized by appearance of MeV-specific IFN-γ-producing CD4^+^ T cells and cytotoxic CD8 ^+^ T cells [40,43,49,50]. However, MeV RNA can be detected in samples from several sites for at least 3 months in naturally infected children and in the lymphoid tissue of experimentally infected macaques for at least six months [37^••^,^43^,^51^,^52^]. The later shift from MeV-specific Th1 to Th2, Th17 and Tfh CD4^+^ T cell responses may be important for the production and maturation of MeV-specific antibodies.

## Maturation of the immune response and development of protective immunity

Protective immunity following WT MeV infection is lifelong [7] as is the production of MeV-specific antibody [8]. Several lines of evidence suggest a primary role for antibody in protection: Vaccine-induced neutralizing antibody levels correlate with protection from clinical measles, passively acquired maternal antibodies protect infants from infection and anti-MeV immune globulin protects exposed, susceptible individuals from disease [53,54]. However, analysis of protection provided by a variety of experimental vaccines in macaques indicates that neutralizing antibody alone generally protects from disease (rash), but not necessarily from infection [55,56]. Vaccine-induced T cell responses alone do not prevent infection or affect the level of viremia or development of a rash, but do lead to more rapid clearance of viral RNA from PBMCs [57].

The mechanism(s) involved in sustaining high levels of neutralizing antibody to MeV are not completely understood, but persistent MeV RNA in lymphoid tissue is associated with continued development of germinal centers, production of ASCs that traffic to the bone marrow and progressive avidity maturation suggesting that prolonged immune stimulation may be an important factor [37^••^]. Immunologic memory to MeV includes both continued circulation of MeV-specific CD4^+^ and CD8^+^ T lymphocytes so long-lasting cellular immunity may also play an important supportive role in protection from infection and disease.

## Immune suppression and effects on pre-existing immune responses

Measles poses an interesting paradox in the juxtaposition of a robust MeV-specific immune response with prominent suppression of immunity to other pathogens and increase in susceptibility to other infectious diseases [58,59^•^]. Most measles deaths are due to other infections [3] and there is an epidemiologically detectable long-term increase in susceptibility to infectious diseases in those who survive [4,5^•^,59^•^]. In addition to infection of B and T cells and a transient lymphopenia during the acute phase of infection, MeV acts both directly on T cells to inhibit activation-induced proliferation and produces a long-term generalized effect on pathogen recognition.

MeV affects T cell function directly by interaction of MeV H and proteolytically activated F with lipid rafts on the cell surface of lymphocytes to block cell cycle progression independent of interaction with SLAMF1. This process involves activation of sphingomyelinases to produce ceramide and inhibit cytoskeletal reorganization required for T cell responses to receptor crosslinking [60, 61, 62, 63].

Measles also increases long-term susceptibility to infection by altering the repertoire of pathogen-specific antibody responses and types of cells in circulation. Numbers of memory B cells are reduced and there is an incomplete reconstitution of naïve B cells [31^•^,64^••^]. In addition, diversity of anti-pathogen antibodies in plasma is decreased presumably due to MeV-induced loss of long-lived plasma cells that produce most of the antibody found in circulation, either due directly to MeV-induced death or eviction from bone marrow niches during the response to MeV [65^••^]. Myeloma cells are susceptible to MeV-induced apoptosis [66], but bone marrow is not a major site of MeV replication [10,37^••^], so the mechanism of plasma cell functional loss during measles requires further study.

## Conclusions

Measles has a complex interaction with the immune system through direct infection of B and T lymphocytes expressing CD150 that results in transient lymphocyte depletion followed by immune activation that generates life-long immunity to reinfection. Over months after apparent recovery and clearance of infectious virus, MeV RNA persists in lymphoid tissue and drives continued stimulation of MeV-specific B and T cell responses. T cells change from IFN-γ production to IL-17 production and numbers of peripheral Tfh cells increase. Germinal centers proliferate and produce antibody-secreting cells that home to the bone marrow and sustain plasma levels of MeV antibody for life. However, levels of antibody to other pathogens decrease and this likely contributes to a long-term increase in susceptibility to other infections.

## Conflict of interest statement

Diane Griffin is a member of the GlaxoSmithKline Vaccines Research Advisory Board and Takeda Pharmaceuticals Zika Vaccine Data Monitoring Committee.

## References and recommended reading

Papers of particular interest, published within the period of review, have been highlighted as

• of special interest
•• of outstanding interest
